# Characterization of immune features and immunotherapy response in subtypes of hepatocellular carcinoma based on mitophagy

**DOI:** 10.3389/fimmu.2022.966167

**Published:** 2022-10-11

**Authors:** Yanan Wang, Boshizhang Peng, Chun Ning, Shuya He, Huayu Yang, Yilei Mao, Lejia Sun

**Affiliations:** ^1^ State Key Laboratory of Medical Molecular Biology, Department of Physiology, Institute of Basic Medical Sciences, Chinese Academy of Medical Sciences and School of Basic Medicine, Peking Union Medical College, Beijing, China; ^2^ Peking Union Medical College and Chinese Academy of Medical Sciences, Beijing, China; ^3^ Department of Physiology, Institute of Basic Medical Sciences, Chinese Academy of Medical Sciences and School of Basic Medicine, Peking Union Medical College, Beijing, China; ^4^ Department of Liver Surgery, Peking Union Medical College (PUMC) Hospital, Peking Union Medical College and Chinese Academy of Medical Sciences, Beijing, China; ^5^ Department of General Surgery, The First Affiliated Hospital, Nanjing Medical University, Nanjing, China; ^6^ The First School of Clinical Medicine, Nanjing Medical University, Nanjing, China

**Keywords:** hepatocellular carcinoma, mitophagy, immune, immune-checkpoint, immunotherapy, prognosis

## Abstract

Mitophagy is suggested to be involved in tumor initiation and development; however, mitophagy heterogeneity in hepatocellular carcinoma (HCC) and its association with immune status and prognosis remain unclear. Differentially expressed genes (DEGs) were identified using expression profiles acquired from The Cancer Genome Atlas (TCGA). Mitophagy-related subtypes were identified using the ConsensusClusterPlus software. The differences in prognosis, clinical characteristics, and immune status, including immune cell infiltration, immune function, immune-checkpoint gene expression, and response to immunotherapy, were compared between subtypes. A mitophagy-related gene signature was constructed by applying least absolute shrinkage and selection operator regression to the TCGA cohort. The International Cancer Genome Consortium cohort and the cohort from Peking Union Medical College Hospital were utilized for validation. Carbonyl cyanide m-chlorophenylhydrazone was used to induce mitophagy in HCC cell lines to obtain our own mitophagy signature. Real-time polymerase chain reaction was used for the experimental validation of the expression of model genes. Two mitophagy-related subtypes with distinct prognoses, clinical characteristics, immune states, and biological function patterns were identified based on the mitophagy-related DEGs. The subtype that showed higher mitophagy-related DEG expression had worse survival outcomes, suppressed immune function, higher immune-checkpoint gene expression, and a better response to immunotherapy, indicating that this subpopulation in HCC may benefit from immune-checkpoint blockade therapy and other immunotherapies. A risk model consisting of nine mitophagy-related genes was constructed and its performance was confirmed in two validation cohorts. The risk score was an independent risk factor even when age, sex, and tumor stage were considered. Our study identified two distinct mitophagy subtypes and built a mitophagy signature, uncovering mitophagy heterogeneity in HCC and its association with immune status and prognosis. These findings shed light on the treatment of HCC, especially with immunotherapy.

## Introduction

Hepatocellular carcinoma (HCC) accounts for the majority of primary liver cancer cases, which is ranked the fifth in cancer-related death (1, 2). Despite much progress in the diagnosis and treatment of HCC, the prognosis of patients with HCC remains poor, with a median survival time of 9 months (3). For patients with HCC at early stage, curative treatments such as radiofrequency ablation and liver section can achieve a 40%-70% 5-year survival rate; and palliative treatments such as transarterial chemoembolization has been shown to improve median OS of intermediate stage HCC to approximately 20 months (4). For HCC at advanced stage or terminal stage, survival outcomes are still unsatisfactory even with the help of molecular therapy. Immunotherapies, such as immune-checkpoint blockade (ICB), have shown strong antitumor activity and lead to a substantial prolonged survival for advanced HCC, whereas only a subset of patients can benefit from these therapies (5). Therefore, there is an urgent need to explore the underlying molecular mechanisms of HCC and provide new targets and strategies for treatment.

Major breakthroughs in a mechanism called mitophagy have recently gained considerable attention (6). Mitophagy, also known as mitochondrial autophagy, eliminates denatured or damaged mitochondria, preventing the accumulation of mitochondrial DNA mutations and maintaining mitochondrial quality (7). Hence, mitophagy plays a vital role in regulating energy metabolism and removing excessive cytotoxic reactive oxygen species (8). Mitophagy plays a dual role in the development of cancer by suppressing tumors at an early stage and promoting tumors at an advanced stage (9). The ubiquitin-dependent PINK1/Parkin pathway is the most common mitophagy cascade, and some core genes within this pathway, such as *PINK1* and *PARK2*, can predict prognosis in patients with papillary renal cell cancer (10, 11). However, the role of mitophagy-related genes (MRGs) in HCC is not fully understood. Several studies have reported heterogeneity in autophagy in other types of cancer (12, 13). As a specific form of autophagy, the heterogeneity of mitophagy likely influences the development and prognosis of HCC. Research focused on mitophagy may help to concretize problems. Therefore, we aimed to investigate the role of MRGs and mitophagy-related subtypes in HCC, focusing on the association with immune status and response to immunotherapy, as there has been massive interest in immunotherapy for HCC (14).

**Figure 1:**
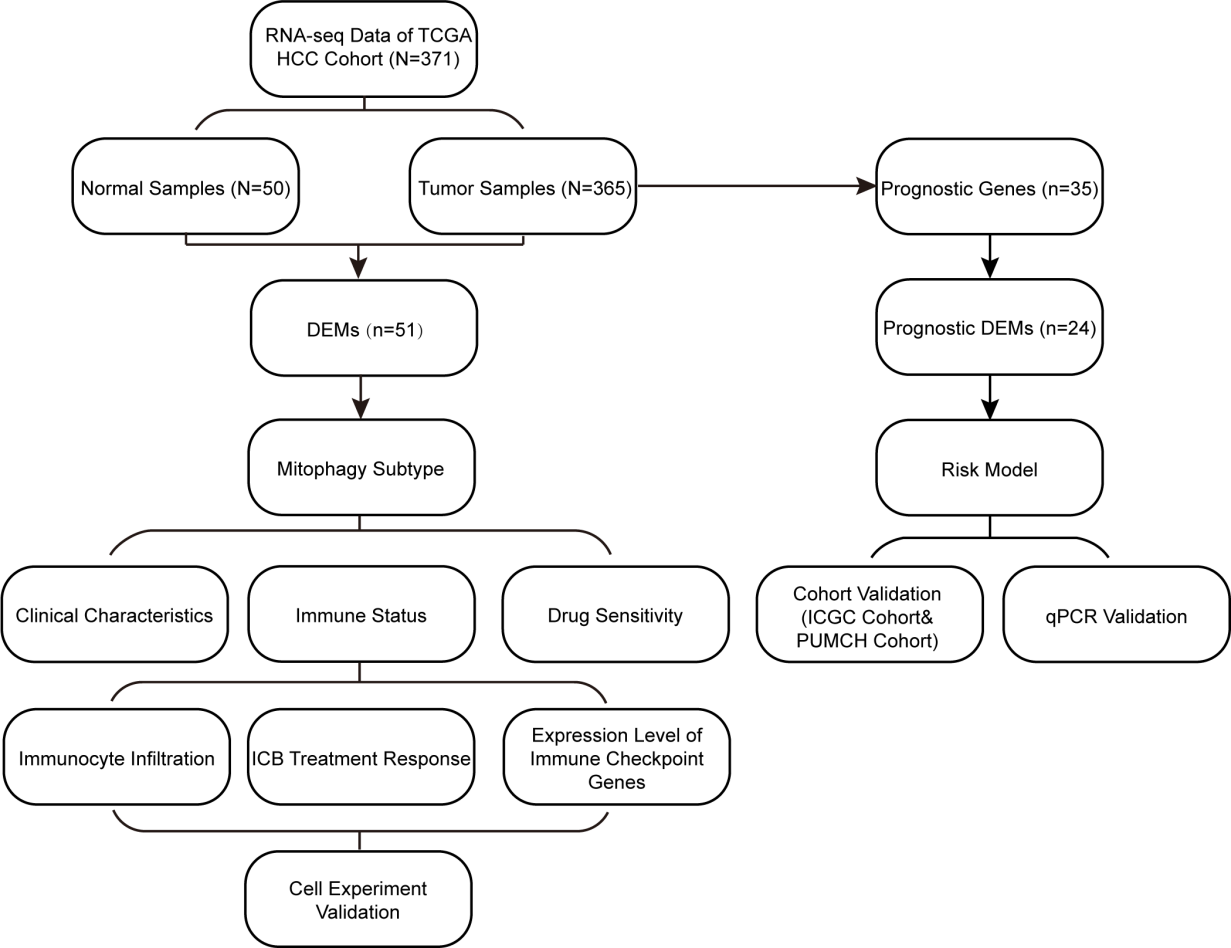
Study flowchart. DEMs, Differentially expressed mitophagy-related genes; ICB, immune-checkepoint blockade.

A flowchart of the study design is shown in **Figure 1**. In this study, we first screened differentially expressed MRGs (DEMs) between tumor and normal tissues of patients with HCC. Based on the expression profile of the DEMs, we classified the patients into two subtypes and explored their prognoses, clinical characteristics, immune states, and drug sensitivities. Subsequently, based on the MRG signature, a prognostic model was constructed and validated in two cohorts. Moreover, we explored the differences in biological functions between these subtypes and risk groups. Cell experiment and qPCR were performed to validate our results. Our findings are helpful in accurately characterizing HCC and providing personalized treatment for patients.

## Materials and methods

### Data acquisition

We obtained two gene sets from public databases by searching the keyword “mitophagy”: one is Mitophagy-animal pathway (Entry: hsa04137), which contains 72 genes from the Kyoto Encyclopedia of Genes and Genomes (KEGG) pathway database (https://www.kegg.jp/kegg/pathway.html), the other one is REACTOME-MITOPHAGY gene sets (source: R-HSA-5205647), which contains 29 genes from the C2:CP : REACTOME in Molecular Signatures Database with Gene Set Enrichment Analysis (GSEA) (http://www.gsea-msigdb.org). Since 20 genes overlapped in two gene sets, we eventually acquired a total of 81 MRGs for subsequent analyses. The RNA-seq and clinical information of HCC samples were downloaded from the Cancer Genome Atlas (TCGA) database (https://portal.gdc.cancer.gov/), which included 50 normal samples and 374 cancer samples. We also acquired two HCC cohort datasets for validation: one was downloaded from the International Cancer Genome Consortium (ICGC) database (https://dcc.icgc.org/), which included 243 cancer samples; the other was collected from Peking Union Medical College Hospital (PUMCH) and included 20 patients with HCC (**Supplementary Table 1**). The cohort from our center was approved by the Ethics Committee of PUMCH and CAMS (Chinese Academy of Medical Sciences) & PUMC (Peking Union Medical College), and written informed consent was obtained from all patients.

### Identification and analysis of DEMs

DEMs between tumor tissues and normal tissues in TCGA were screened using the “limma” R package (15) based on the following criteria: |log_2_fold change| > 0.5 and false discovery rate < 0.05. The protein-protein interaction network of DEMs was obtained from the Search Tool for the Retrieval of Interacting Genes/Proteins (STRING) database (https://string-db.org), and the interaction between core genes was visualized using Cytoscape software (version 3.8.2) (16).

### Consensus cluster analysis

We used the ConsensusClusterPlus software (17) of R to perform unsupervised consensus clustering of TCGA dataset based on the expression of DEMs. The optimal cluster number *k* was determined by evaluating the delta area, consensus cumulative distribution function, and consensus matrix. Principal component analysis was used to verify the results of the cluster analysis. The correlation between clusters and clinical variables was tested using Chi-square test.

### Immune status analysis

To explore the impact of mitophagy on patient immune status, two mitophagy-related subtypes were compared in terms of differences in infiltrating immune cells, immune function, immune-checkpoint gene expression levels, and response to immunotherapy. We quantified the relative abundance of immune cell types and the activity of immune function in each sample using single sample GSEA algorithms through the R package “GSVA” (18). The expression levels of immune-checkpoint genes can reflect the response to ICB treatment; thus, the following well-known immune-checkpoint genes were chosen for expression level evaluation in each subtype: *CTLA4*, *CXCL9*, *CD8A*, *TBX2*, *PDCD1*, *LAG3*, *HAVCR2*, *IFNG*, *TNF*, and *CD274*. Moreover, Tumor Immune Dysfunction and Exclusion (TIDE) scores (19) of each HCC sample were calculated online (http://tide.dfci.harvard.edu/) and compared between subtypes to verify the differences in response to immunotherapy.

### Drug sensitivity analysis

To discover potential drugs for patients with different mitophagy-related subtypes of HCC, we evaluated their responses to various antitumor drugs using the “pRRophetic” R package (20), which is based on the Genomics of Drug Sensitivity in Cancer database.

### Construction and validation of risk model

First, we performed univariate Cox regression analysis on MRGs to screen for survival-related prognostic genes in the TCGA cohort. We then obtained genes for model construction by intersecting prognostic genes with DEMs, followed by least absolute shrinkage and selection operator (LASSO) regression using the “glmnet” R package (21) to form the final gene signature for the risk model. The risk score was formulated as follows:


risk score = β1 × gene1 expression + β2 × gene2 expression +  …  + βn × gene expression

where β represents the coefficient value of each gene. Patients in the training and validation cohorts were divided into high- and low-risk groups based on the median risk scores. The receiver operating characteristic (ROC) curves in each cohort were plotted using the R package “timeROC” (22), and the time-dependent area under the curve values were measured to evaluate the performance of the model. Univariate and multivariate Cox regression analyses were used to evaluate whether the risk score was an independent predictor.

### Functional analysis

KEGG and Gene Ontology (GO) enrichment analyses were utilized for functional annotation of DEMs and differentially expressed genes (DEGs) between subtypes using the “ClusterProfiler” R package (23). Significant GO terms and pathways were selected with a *p*-value cutoff of < 0.01. The biological functions enriched in the high- and low-risk groups of the TCGA cohort were derived by GSEA of KEGG pathways.

### Acquisition of mitophagy signature by inducing mitophagy in HCC cell lines

Human HCC cell line Huh 7 was purchased from Procell Life Science & Technology Co. Ltd. (Wuhan, China). MHCC97H was from the Liver Cancer Institute (Zhongshan Hospital, Fudan University, China). SNU398 was from ATCC (Manassas, VA, USA). Huh7 and MHCC97H were cultured in Dulbecco’s modified Eagle medium, supplemented with 10% fetal bovine serum and 1% antibiotics in 5% CO_2_ at 37°C. SNU398 was routinely cultured in RPMI-1640 medium supplemented with 10% fetal bovine serum and 1% antibiotics in 5% CO_2_ at 37°C. Then three HCC cell lines were treated with 10μM carbonyl cyanide m-chlorophenylhydrazone (CCCP, selleck,China), which was used to induce mitophagy, for 24 hours (24, 25). RNA sequencing for Huh 7, MHCC97H, SNU398 cells treated with or without CCCP by Beijing Auwigene Tech, Ltd (Beijing, China) using the Illumina second-generation high-throughput PE150 sequencing platform (Illumina, Inc., CA, United States). Between cell lines treated with and without CCCP, top 100 differentially expressed genes ranked by |log_2_fold change| were considered as mitophagy signature for validation.

### Real-time polymerase chain reaction

Total RNA was extracted from liver tumors and peritumoral tissues using TRIzol (Invitrogen, Thermo Fisher, Waltham, MA, USA), following the manufacturer’s instructions. RNA (1 µg) was reverse transcribed using the Hifair^®^ II 1st Strand cDNA Synthesis SuperMix for qPCR (gDNA digester plus) (Yeasen Biotechnology, Shanghai, China) in a 20 μl reaction. After 20-fold dilution, 4 μl of cDNA was used as a template in a 20 μl real-time polymerase chain reaction (PCR). For real-time PCR, amplification was performed for 40 cycles using BlasTaq™ 2X qPCR MasterMix (Applied Biological Materials Inc., Richmond, BC, Canada). Primers were designed on exon junctions to prevent co-amplification of genomic cDNA; the sequences are presented in the **Supplementary Table 2**.

### Statistical analyses

All statistical analyses were performed using R (version 4.0), and relevant packages were applied for processing and visualization. The Wilcoxon test was used to compare differences in continuous variables. The overall survival was evaluated using the Kaplan–Meier analysis, and the survival curve was plotted using the R package “survminer” (http://cran.r-project.org/). The log-rank test was used to examine the differences between subtypes or groups. If not specifically stated, bilateral *p* < 0.05 was considered statistically significant.

## Results

### Profile and functional annotation of DEMs between tumor and normal samples

**Figure 2:**
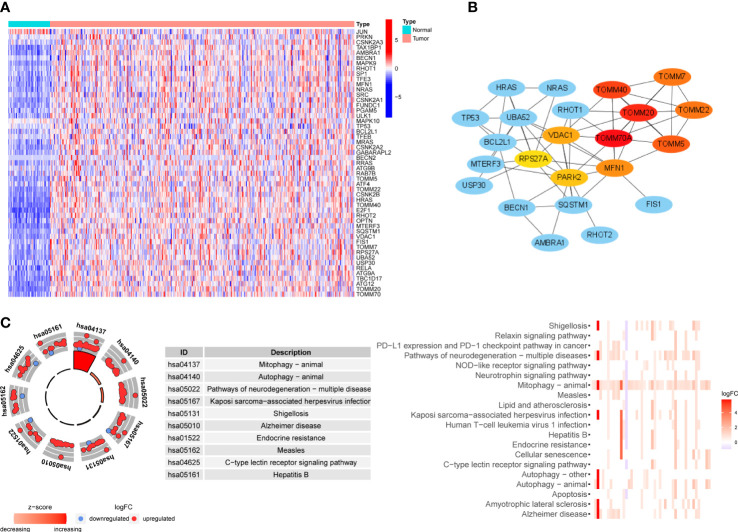
Expression level, interactions and functional enrichment analysis of DEMs between tumor samples and normal samples. **(A)** Profile of DEMs based on sample type. Color represents expression level (blue to red). **(B)** Hub protein-protein interaction network among DEMs. Color represents confidence (blue to red). **(C)** KEGG analysis of DEMs. DEMs: Differentially expressed mitophagy-related genes, KEGG: Kyoto Encyclopedia of Gene and Genome.

We collected 81 MRGs from the database gene sets, and 51 DEMs were identified between tumor and normal samples from the TCGA cohort. In order to verify DEMs acquired from one public database, we performed RNA sequencing on 8 pairs of HCC samples and peritumoral tissues, and the expression matrix can be found in **Supplementary Table 1**. Of 42 DEMs identified in our own samples, 35 DEMs overlapped with 51 DEMs from TCGA cohort, and 39 of 40 DEMs identified in ICGC cohort overlapped with those in TCGA cohort, indicating the reliability of DEMs identified in TCGA cohort. A heatmap of the 51 DEMs is shown in **Figure 2A**. Fifty of the DEMs were upregulated in tumors, primarily including genes involved in the PINK1/Parkin pathway (*PINK1*, *PARK2*, *ATG* family, and *TOMM* family) and receptor-mediated mitophagy (*FUNDC1*, *PGAM5*, and *ULK1*). In addition, oncogenes such as *TP53*, *KRAS*, and *HRAS* were also upregulated, as these genes may be related to hypoxic stress. Only one gene, *JUN*, was downregulated. We then performed protein-protein interaction analysis to determine the interactions between DEMs and the core network, as shown in **Figure 2B**; the *TOMM* family, *MFN1*, *VDAC1*, *PARK2*, and *RPS27A* were hub genes, and oncogenes such as *HRAS*, *KRAS*, and *TP53* participated in the core network of mitophagy. The KEGG analysis in **Figure 2C** showed that, besides mitophagy and autophagy, these DEMs were also enriched in the PD-1/PD-L1 checkpoint pathway, which is related to the response to ICB treatment of HCC. Pathways involving hepatitis B and apoptosis were also enriched, and they were shown to be involved in tumorigenesis and the development of HCC (26, 27).

### Subpopulation of HCC on account of expression pattern of DEMs

**Figure 3:**
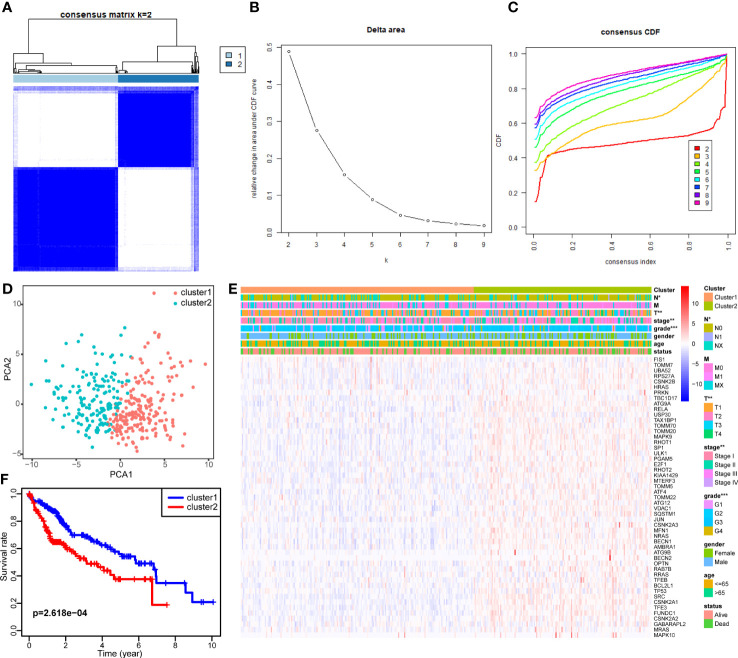
Identification of two mitophagy-related HCC subtypes with different prognosis. **(A–C)** Consensus matrix of HCC samples co-occurrence proportion for k = 2 **(A)**, relative change in area under the CDF curve for k from 2 to 7 **(B)**, consensus clustering CDF for k from 2 to 7 **(C)**. **(D)** Principal component analysis of HCC samples grouped by subtype. **(E)** A heatmap showing the association of mitophagy-related subtypes with clinical characteristics and expression of DEMs. **(F)** The Kaplan–Meier plot showing the overall survival differences between the two subtypes. The asterisks represent the *p* value (*p < 0.05; **p < 0.01; ***p < 0.001). DEMs, Differentially expressed mitophagy-related genes. CDF, Cumulative distribution function.

Consensus clustering was applied to identify HCC subtypes based on the expression levels of the DEMs acquired from the previous step. We determined the *k* value as 2, at which point the relative change in area under the cumulative distribution function reached an approximate maximum and the consensus matrix showed a clear boundary simultaneously (**Figures 3A–C**). Therefore, two clearly distributed subtypes were classified; these were denoted as cluster 1 (containing 211 samples) and cluster 2 (containing 163 samples). To further verify the clustering result, principal component analysis was performed, and the principal component distribution was in accordance with the consensus matrix, ensuring the stability of consensus clustering (**Figure 3D**). The DEM expression and clinical features of each sample grouped by cluster are shown in a heat map (**Figure 3E**). Cluster 2 generally had higher DEM expression than cluster 1. Tumor stage and grade were found to be correlated with subtype. Cluster 2 had a higher proportion of tumors with advanced stage and high grade. Moreover, the Kaplan–Meier survival analysis showed that the two subtypes had significant differences in OS (**Figure 3F**). Cluster 2 tended to have worse outcomes than cluster 1 (*p* = 2.618e-04), with 5-year survival rates of 37.6% and 56.0%, respectively. The difference in prognosis between the two clusters was in accordance with their differences in tumor stage and grade. These findings confirm the existence of mitophagy heterogeneity in HCC and its impact on the development and prognosis of HCC.

### Characterization of immune status and drug sensitivity affected by mitophagy heterogeneity in HCC

**Figure 4:**
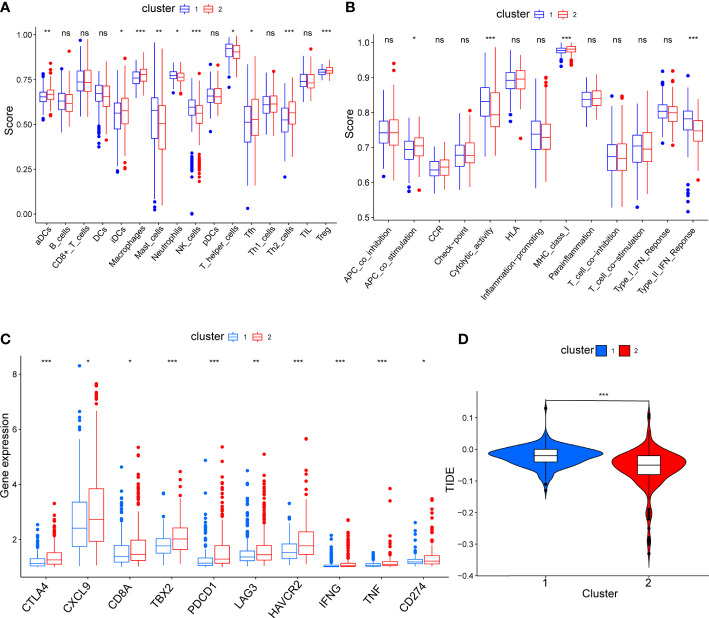
The comparison of immune status between mitophagy-related subtypes. Box plots showing the differences of infiltrating immunocyte abundance **(A)**, immune reaction activity **(B)**, expression of immune-checkpoint genes **(C)** and violin plots showing Tumor Immune dysfunction and Exclusion (TIDE) score **(D)**. The asterisks represent the *p* value (*p < 0.05; **p < 0.01; ***p < 0.001). ns, not significant.

To compare the immune characteristics of the two mitophagy-related subtypes, we first estimated immunocyte infiltration and immune function using single sample GSEA algorithms. As shown in **Figure 4A**, compared with cluster 1, cluster 2 showed higher infiltration of activated dendritic cells (aDCs), immature dendritic cells (iDCs), macrophages, follicular helper T cell (Tfh), T helper 2 cell (Th2) and regulatory T cells (Treg). Regarding immune function in **Figure 4B**, cytolytic activity and type II interferon response were increased in cluster 1, while cluster 2 had strong antigen-presenting cell (APC) co-stimulation and major histocompatibility complex (MHC) class I reactions.

To explore the effect of mitophagy on the response to ICB treatment, we compared the expression levels of immune-checkpoint genes in each subtype. As shown in **Figure 4C**, all immune-checkpoint genes were consistently overexpressed in cluster 2, indicating that cluster 2 tended to be more sensitive to ICB treatment. Furthermore, we calculated the TIDE score of every sample and the scores were significantly lower in cluster 2 than in cluster 1, further verifying that patients in cluster 2 may be more likely to benefit from immunotherapy (**Figure 4D**). In contrast, with higher TIDE scores, cluster 1 was more likely to achieve tumor immune escape and exhibit a lower response rate to ICB treatment.

**Figure 5:**
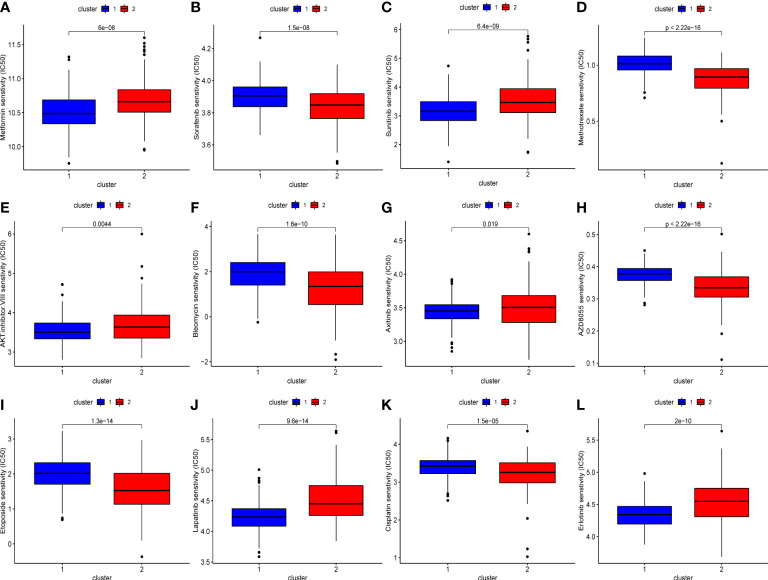
The difference of chemo drugs sensitivity between subtypes, including metformin **(A)**, sorafenib **(B)**, sunitinib **(C)**, methotrexate **(D)**, AKT.inhibitor.VIII **(E)**, bleomycin **(F)**, axitinib **(G)**, AZD8055 **(H)**, etoposide **(I)**, lapatinib **(J)**, cisplatin **(K)**, erlotinib **(L)**.

We also evaluated the drug sensitivity of each subtype to identify potential chemotherapeutic drugs. Lower IC50 values indicate higher sensitivity. As shown in **Figure 5**, compared with cluster 2, cluster 1 was more sensitive to AKT inhibitor III, epidermal growth factor receptor inhibitors such as erlotinib, gefitinib, and lapatinib, and vascular endothelial growth factor receptor inhibitors such as axitinib and sunitinib. Conversely, cluster 2 had a higher response rate to AZD8055 (mTOR inhibitor), bleomycin, cisplatin, etoposide, sorafenib, and methotrexate.

### Functional annotation of DEGs between subtypes

**Figure 6:**
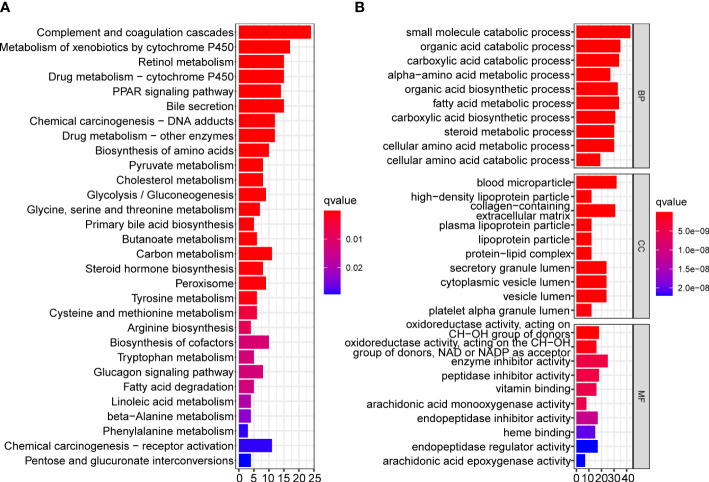
Functional enrichment analysis of DEGs between two mitophagy-related subtypes. Bar plots showing the biological function of DEGs using KEGG **(A)** and GO **(B)** enrichment. DEGs, Differentially expressed genes; KEGG, Kyoto encyclopedia of genes and genomes; GO, Gene ontology.

To reveal the differences in biological functions between the two subtypes, we conducted GO and KEGG enrichment analysis on the DEGs between the two subtypes with a cutoff of |log_2_fold change|> 1 and false discovery rate < 0.05. A total of 260 DEGs met the criteria, and the results showed that complement and coagulation cascades, the peroxisome proliferators-activated receptors signaling pathway, bile secretion, chemical carcinogenesis, and drug and compound metabolism were significantly enriched in the KEGG pathway analysis (**Figure 6A**). The results of GO enrichment analysis are shown in **Figure 6B**.

### Constructing prognosis model of MRGs

**Figure 7:**
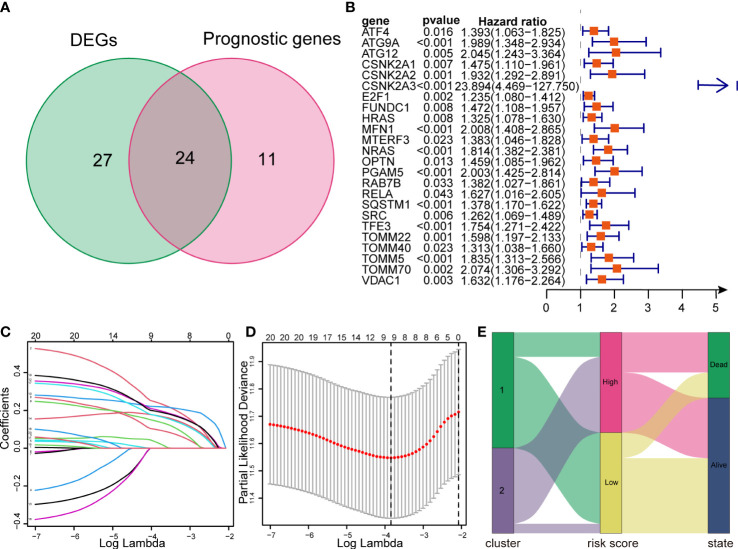
Construction of a LASSO regression model and correlation between subtypes and risk groups. **(A)** Venn diagram showing intersection between DEMs and prognostic genes. **(B)** Forest plots showing the results of univariate Cox regression analysis of overlapped genes. **(C, D)** LASSO regression analysis of the overlapped genes. **(E)** The Sankey diagram showing the distribution of patients in mitophagy-related subtypes, risk groups and survival outcomes. LASSO, Least absolute shrinkage and selection operator.

Defining the TCGA dataset as a training cohort, we performed univariate Cox regression analysis on 81 MRGs, 35 of which were significantly associated with the OS of patients with HCC (*p* < 0.05). After intersection with 51 DEMs, we obtained 24 genes for model construction (**Figure 7A**). All 24 genes were risk genes with a hazard ratio of > 1 (**Figure 7B**). The LASSO regression model was then utilized, and nine genes were screened to build the prognostic risk model (**Figures 7C, D**). The risk score was calculated using the corresponding coefficients and gene expression. Finally, the risk score model was formulated as follows:


$$\begin{array}{l} ATG9A\;\times \;0.2414855\;+ATG12\;\times \;0.0294790\\ \;+HRAS\;\times \;0.0977971\;+MFN1\;\times \;0.1418254\\ \;+NRAS\;\times \;0.2199555\;+PGAM5\;\times \;0.2023505\\ \;+SQSTM1\;\times \;0.1721932\;+TOMM22\\ \times \;0.1709305\;+TOMM5\;\times \;0.1709305 \end{array}$$

Additionally, we created a Sankey diagram to show the connection among mitophagy subtypes, risk scores, and survival (**Figure 7E**).

### Validation of model efficiency

**Figure 8:**
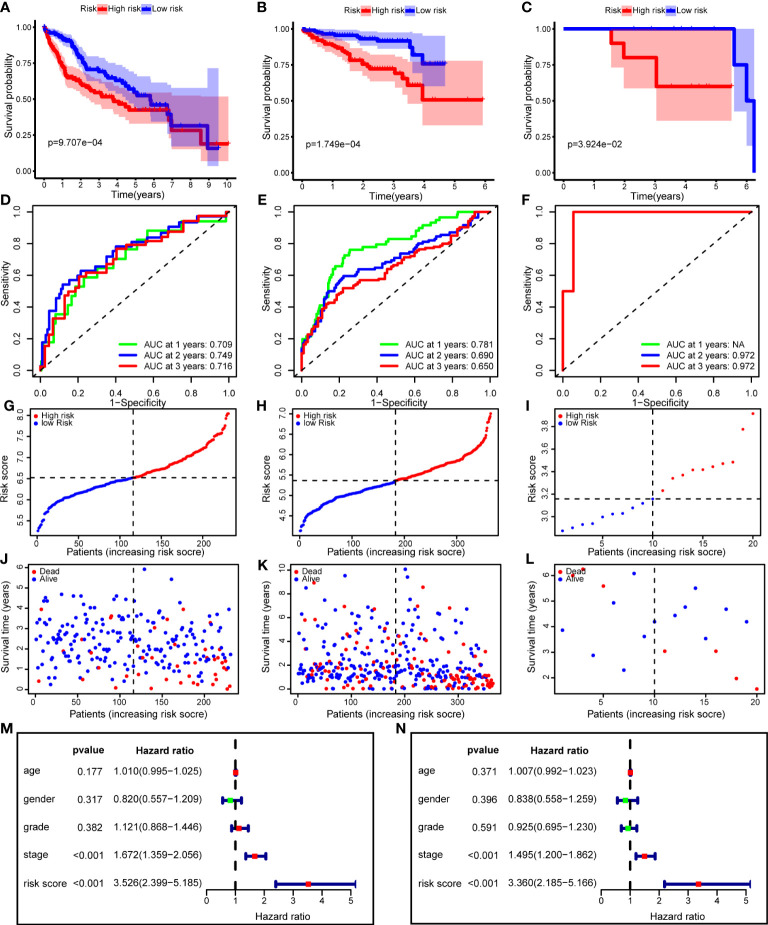
The risk model performance in training cohort and two validation cohorts. **(A–C)** Kaplan-Meier curves for the OS of patients in the high- and low-risk group in the TCGA cohort **(A)**, ICGC cohort **(B)** and PUMCH cohort **(C)**. **(D–F)** AUC of time-dependent ROC curves in the TCGA cohort **(D)**, ICGC cohort **(E)** and PUMCH cohort **(F)**. **(G–I)** The distribution and median value of the risk scores in the TCGA cohort **(G)**, ICGC cohort **(H)** and PUMCH cohort **(I)**. **(J–L)** The distributions of risk scores, survival states and survival outcomes in the TCGA cohort **(J)**, ICGC cohort **(K)** and PUMCH cohort **(L)**. **(M, N)** Forest plots showing the univariate **(M)** and multivariate **(N)** Cox regression analyses regarding OS in the TCGA cohort. OS, Overall survival, AUC, area under the curve, ROC, receiver operating characteristic, TCGA, The Cancer Genome Atlas, ICGC, International Cancer Genome Consortium, PUMCH, Peking Union Medical College Hospital.

To verify the performance of the risk model, we performed Kaplan–Meier survival analysis in the training and validation cohorts. The survival curves showed that improved survival rates of low-risk patients continued for nearly 7 years in the TCGA training cohort (*p* = 9.707e-04), and this advantage existed in the ICGC validation cohort (*p* = 1.749e-04) (**Figures 8A, B**). In addition, we used an HCC cohort (n=20) registered in our center to validate the risk model, and the difference in OS was still significant (*p* = 3.924e-02) (**Figure 8C**). Regarding model accuracy, the 1-year, 3-year, and 5-year AUC of the model for OS was 0.781, 0.690, and 0.650, respectively, in the TCGA training cohort (**Figure 8D**), and 0.709, 0.749, and 0.716, respectively, in the ICGC validation cohort (**Figure 8E**). The AUC of the model in PUMCH cohort was still satisfactory (**Figure 8F**). We ranked the risk scores of patients with HCC in all cohorts from low to high, and the survival status and time of each patient were plotted according to the risk score (**Figures 8G–L**). The plot revealed that high-risk patients generally had poorer survival rates than low-risk patients.

To determine whether the risk score is an independent risk factor for the prognosis of patients with HCC, we performed univariate Cox regression on the risk score and clinical variables (**Figure 8M**). The results showed that only the stage and risk scores were significantly associated with OS (*p* < 0.001). Next, these variables were included in the multivariate Cox regression analysis. After correction for other confounding factors, including age, sex, stage, and grade, the risk score was still significantly associated with OS, implying that the risk score was an independent risk factor (*p* < 0.001) (**Figure 8N**).

### Functional enrichment analysis based on the risk score

**Figure 9:**
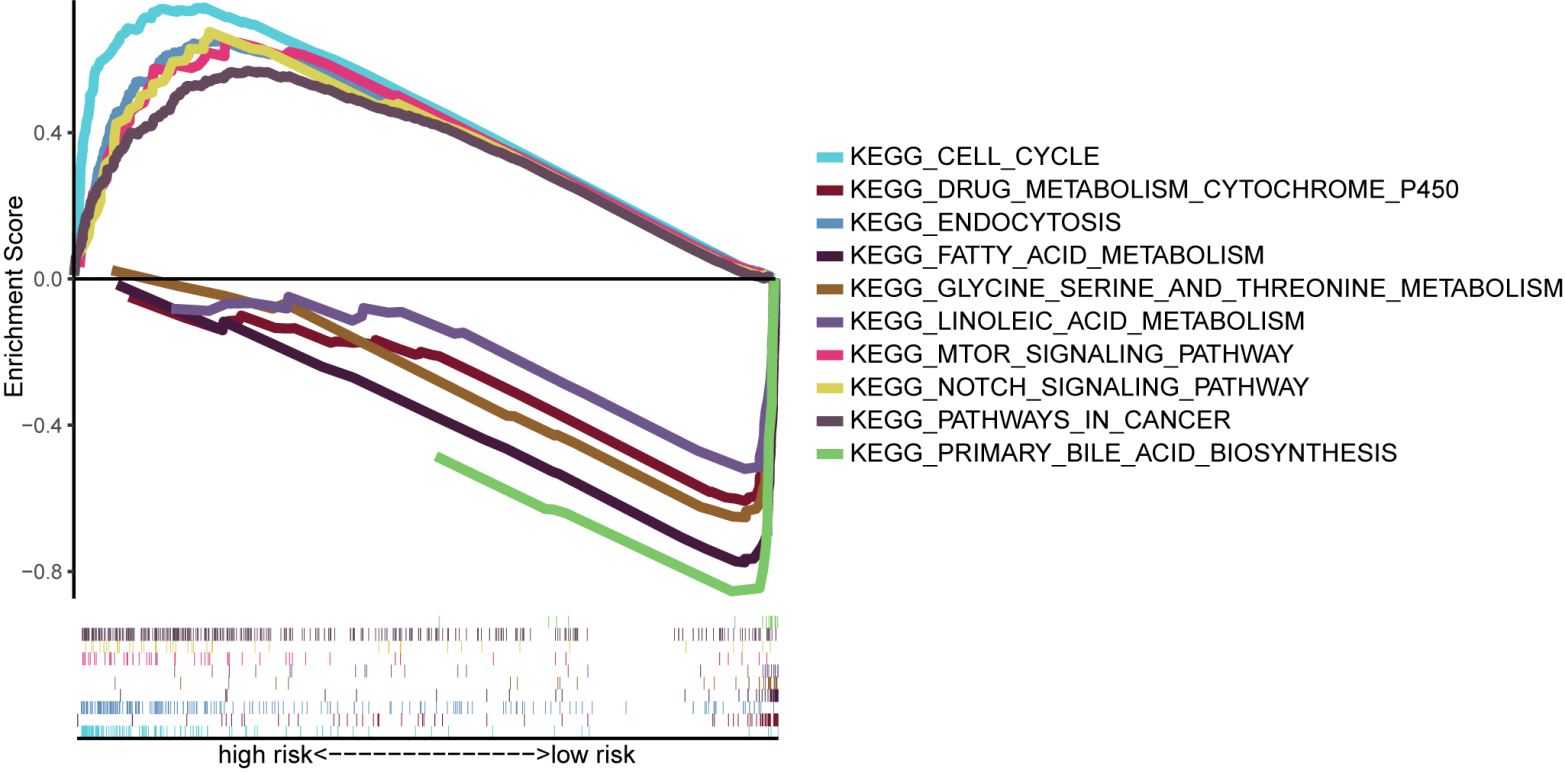
Gene-set enrichment analysis identifying KEGG pathways enriched in the high- and low-risk group. KEGG, Kyoto Encyclopedia of Gene and Genome.

We performed GSEA on the TCGA cohort, and the most significantly enriched KEGG pathways are shown in **Figure 9**. The cell cycle, mTOR signaling pathway, NOTCH signaling pathway, endocytosis, and pathways in cancer were enriched in the high-risk group. Primary bile acid biosynthesis, drug metabolism, cytochrome P450, fatty acid metabolism, glycine serine and threonine metabolism, and linoleic acid metabolism pathways were enriched in the low-risk group.

### Validation of mitophagy heterogeneity through cell experiment

**Figure 10:**
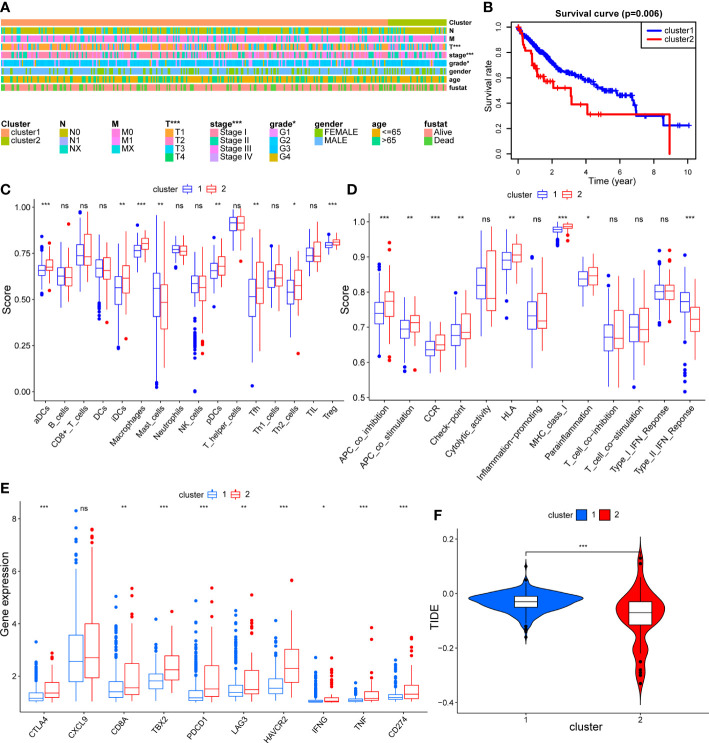
The validation of mitophagy-related HCC subtypes using mitophagy signature obtained from cell experiment. **(A)** A heatmap showing association between subtypes and clinical characteristics. **(B)** The Kaplan–Meier plot showing distinct prognosis between two subtypes. **(C–E)** Box plots showing the differences of infiltrating immunocyte abundance **(C)**, immune reaction activity **(D)**, and expression of immune-checkpoint genes **(E)** between two subtypes. **(F)** Violin plots comparing the Tumor Immune dysfunction and Exclusion (TIDE) score of each subtype. The asterisks represent the *p* value (*p < 0.05; **p < 0.01; ***p < 0.001). ns, not significant.

In order to validate mitophagy-related subtypes obtained using public mitophagy gene sets, we acquired mitophagy signature through inducing mitophagy in HCC cell lines for same analyses in TCGA HCC dataset. The expression matrix of HCC cell lines before and after mitophagy induction was demonstrated in **Supplementary Table 1** and the expression levels of mitophagy signature genes were applied for clustering of TCGA HCC dataset. As shown in **Figure 10A**, tumor grade and stage were still correlated with clusters. And cluster 2 had significantly worse survival outcome than cluster 1 (*p* = 0.006) (**Figure 10B**). Similar to results in **Figure 4A**, cluster 2 had higher infiltration of aDCs, iDCs, macrophages, Tfh, Th2, and Treg than cluster 1 (**Figure 10C**). In terms of immune function, type II interferon response was still suppressed in cluster 2, while check-point, APC co-stimulation, APC co-inhibition, HLA, MHC class I, and proinflammation exhibited higher levels in cluster 2 (**Figure 10D**), which confirmed the association between mitophagy and immune status in HCC. In addition, regarding response to ICB treatment, all immune-checkpoint genes except *CXCL9* were significantly overexpressed in cluster 2 (**Figure 10E**), and TIDE scores remained lower in cluster 2 than in cluster 1 (**Figure 10F**), indicating that patients in cluster 2 tended to benefit from ICB treatment. These findings following cell experiment further validated our results from using public MRGs.

### Validation of expression of model genes in tissue

**Figure 11:**
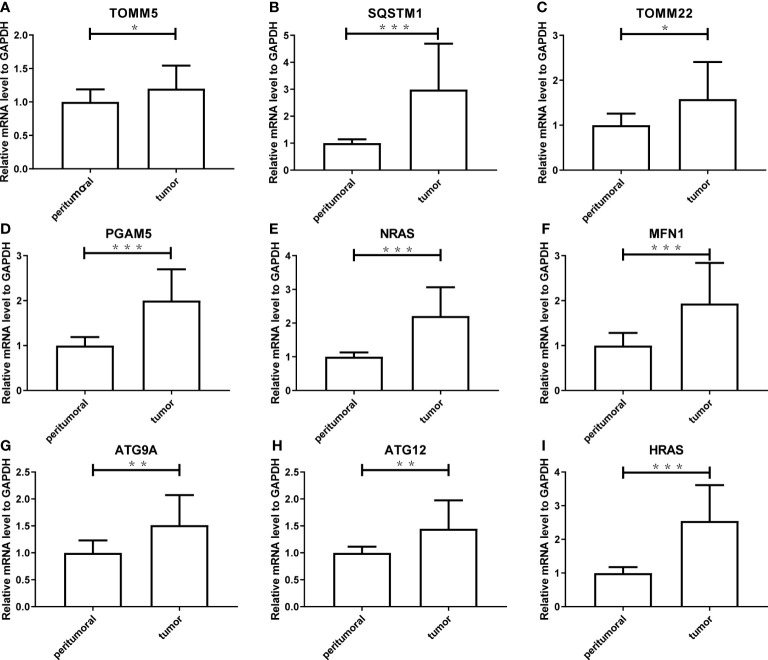
The experimental validation of nine genes consisting the risk model using Real-Time PCR, including *TOMM5* **(A)**, *SQSTM1* **(B)**, *TOMM2* **(C)**, *PGAM5* **(D)**, *NRAS* **(E)**, *MFN1* **(F)**, *ATG9A* **(G)**, *ATG12* **(H)**, *HRAS* **(I)**. The asterisks represent the *p* value (*: *p* < 0.05; **: *p* < 0.01; ***: *p* < 0.001). PCR, polymerase chain reaction.

To verify the reliability of the results acquired from the public database, we further validated the expression levels of the nine genes consisting of the mitophagy signature in five pairs of HCC tissues and peritumoral tissues. As shown in **Figure 11**, the tumor expressed significantly higher mRNA levels of all genes (*ATG9A*, *ATG12*, *HRAS*, *MFN1*, *NRAS*, *PGAM5*, *SQSTM1*, *TOMM22*, and *TOMM5*) than peritumoral tissue, which was consistent with the public database analysis.

## Discussion

Emerging immunotherapy, especially ICB treatment, has become an effective and promising option to treat HCC (28). However, only a portion of patients respond to immunotherapy; thus, it is important to determine which groups of patients can benefit from immunotherapy, facilitating the progress of personalized treatment. Recently, mitophagy has attracted the attention of researchers as a potential therapeutic target for cancer. Hence, this study aimed to investigate mitophagy heterogeneity in HCC and its association with immune status, identify two mitophagy subtypes with distinct clinical and immune characteristics, and offer more detailed insights into immunotherapy or combination therapy for HCC.

HCC has been confirmed to exhibit high molecular heterogeneity (29). We identified two mitophagy subtypes in TCGA HCC samples based on the expression levels of DEMs, showing that these two subtypes with different mitophagy patterns were characterized by significantly different tumor stages and prognoses. This verified that mitophagy heterogeneity is associated with HCC development and has prognostic value in HCC, although the underlying mechanisms are still not well understood. Mitophagy appears to be tumor-promoting or tumor-suppressive, depending on the tumor type and intrinsic stage (30). For instance, *PARK2*, which encodes a core mitophagy protein, Parkin, was found to be inactivated in colon and lung cancer (31). Parkin-null mice are susceptible to spontaneous HCC (32). In contrast, upregulation of the mitochondrial inner membrane protein STOML2 can amplify PINK1/Parkin-mediated mitophagy and facilitate the migration and invasion of HCC cells, thus promoting HCC growth and metastasis (33). Previous studies have also found that hyperactivated mitophagy can induce sorafenib resistance in HCC under hypoxic stress (34). Therefore, the dual role of mitophagy may be involved in HCC heterogeneity. In our study, nearly all DEMs were upregulated in HCC samples compared with normal tissues, and cluster 2 had generally higher expression levels of DEMs but worse prognosis than cluster 1. Based on the above evidence, cluster 2 is likely to be characterized by higher mitophagy activity, which results in a more advanced tumor and worse survival outcome. Furthermore, regulation of various mitophagy pathways, such as PINK1/Parkin-mediated mitophagy and BNIP3/BNIP3L/FUNDC1-mediated mitophagy, may also be involved in HCC heterogeneity, which warrants further study.

To determine whether mitophagy heterogeneity has an impact on the tumor immune microenvironment, we evaluated the differences in immune characteristics between the two subtypes. Immune cell infiltration is closely related to clinical outcomes, and immune cells can serve as an immunotherapy target (35). Our single sample GSEA results indicated that cluster 2 had a higher abundance of regulatory T cells and macrophages, which are considered to be HCC promoting (36). Moreover, cluster 2 was characterized by higher expression levels of immune-checkpoint genes. Overexpression of immune-checkpoint genes can suppress the antitumor immune response so that tumor cells can easily evade immune surveillance. These findings explain the poor survival outcomes of cluster 2.

ICB therapy can restore dysfunctional immune system and has achieved remarkable results in cancer treatment. ICB agents against programmed cell death protein 1 (PD-1) and cytotoxic T lymphocyte antigen 4 (CTLA-4) have been approved for HCC by the FDA (37). However, the limited response rate makes it important to screen patients who are sensitive to ICB therapy. Cluster 2 showed higher expression levels of immune check-point genes, indicating a better response to ICB treatment. The TIDE algorithm is believed to perform better than the expression level of immune check-points in predicting the survival outcome of cancer patients treated with ICB agents (19). Corresponding to the prediction based on immune-checkpoint expression, the TIDE results revealed that cluster 2 was more likely to respond to ICB treatment. Therefore, ICB treatment may help reverse the poor prognosis of cluster 2. Taken together, mitophagy heterogeneity in HCC may influence immune status and can predict the response rate to ICB agents, revealing the association between mitophagy and immunity. This result enhanced our understanding of the heterogeneity of HCC, promoting personalized therapy in clinical practice and inspiring immunotherapy development in scientific research and trials. Furthermore, our study explored potential drugs for subpopulations with different mitophagy patterns, providing ideas for synergistic combination of ICB agents and targeted therapies. Systemic therapy in HCC should be explored to improve clinical efficacy (38).

To more precisely predict the prognosis of patients with HCC, we constructed a risk model based on a mitophagy signature. Notably, all nine genes were risk factors for HCC. Of these genes, *ATG9A* and *ATG12* are core regulators of autophagy (39). *MFN1*, also known as mitofusin-1, was analyzed both *in vivo* and *in vitro* and its effects on HCC metastasis were revealed (40). *PGAM5* is an atypical mitochondrial serine/threonine phosphatase that dephosphorylates *FUNDC1* to activate mitophagy. Previous studies have reported that depleting *PGAM5* inhibits tumor development and enhances the 5-fluorouracil sensitivity of HCC cells (41, 42). The TOMM complex (translocase of the outer mitochondrial membrane) imports nearly all mitochondrial proteins from the cytoplasm into the mitochondria, and TOMM22 functions as a central receptor (43). Although no significant increase in the expression of *TOMM* genes was observed in prostate cancer compared to normal tissues, our results demonstrated that this protein was elevated in HCC and may be a good candidate biomarker; this requires validation (44). Through ROC analysis in different cohorts, we found that our risk model showed better efficacy in predicting prognosis compared to models constructed based on other gene signatures, such as pyroptosis in HCC (45). Remarkably, the performance of the model was consistently stable and even better in the validation cohort of ICGC and our own cohort than in the training cohort, which verifies the robustness of our risk model.

Functional analyses revealed that various metabolic pathways were enriched in the mitophagy subgroups and risk groups. Metabolic reprogramming is a hallmark of tumor growth and progression (46). Mitophagy plays a critical role in the metabolic adaptation of cancer cells so that these cells can survive under stress factors produced in the tumor microenvironment, and these adaptions are closely related to the acquisition of metastatic potential and chemoresistance (47). Therefore, some metabolic regulators or pathways related to mitophagy may serve as new therapeutic targets for cancer. Additionally, metabolic pathway regulation can affect immune cell function and fate, leading to a connection to the immune microenvironment (48). This crosstalk between metabolic reprogramming and the immune microenvironment adds further layers to the search for novel therapeutic strategies, regardless of forthcoming challenges. Combining existing evidence and our results, we hypothesize that a metabolism-mitophagy-immunity network exists in HCC, which needs to be explored and validated in future studies.

Nevertheless, our study has several limitations. First, our study focused on the expression of genes, lacking multi-omics data, such as copy number variations and DNA methylation. Second, the study was conducted retrospectively based on data from a public database rather than using a prospective cohort. Furthermore, HCC cell lines and our own HCC cohort used for validation had limited sample sizes, though the results are still reliable. Finally, the mechanisms underlying mitophagy, metabolism, and immunity in tumors warrant further study.

## Conclusion

In summary, we identified two prognostically and clinically relevant mitophagy subtypes in HCC. These two subtypes differed in multiple aspects, including immune characteristics, responses to immunotherapy, and biological functions. We also constructed a mitophagy-related risk model that exhibited stable efficiency and performed better than models based on other signatures. The expression of these model genes was subsequently validated using laboratory results. These findings suggest mitophagy as a potential treatment target and shed new light on the strategy of immunotherapy in HCC.

## Data availability statement

The datasets presented in this study can be found in online repositories. The names of the repository/repositories and accession number(s) can be found in the article/**Supplementary Material**.

## Ethics statement

The studies involving human participants were reviewed and approved by ethics committee of PUMCH and CAMS (Chinese Academy of Medical Sciences) & PUMC (Peking Union Medical College). The patients/participants provided their written informed consent to participate in this study.

## Author contributions

LS designed the study. YW, BP, and CN performed the statistical analysis and wrote the first manuscript. YW and SH performed laboratory experiment. YM and HY collected the data and provided the specimen. LS revised the manuscript. All authors contributed to the article and approved the submitted version.

## Funding

This work was supported by National Natural Science Foundation of China (81602456, 81930053).

## Acknowledgments

We are sincerely acknowledged the contributions from the TCGA project and the ICGC project.

## Conflict of interest

The authors declare that the research was conducted in the absence of any commercial or financial relationships that could be construed as a potential conflict of interest.

## Publisher’s note

All claims expressed in this article are solely those of the authors and do not necessarily represent those of their affiliated organizations, or those of the publisher, the editors and the reviewers. Any product that may be evaluated in this article, or claim that may be made by its manufacturer, is not guaranteed or endorsed by the publisher.
