# Supplementary material for: Characterization of immune features and immunotherapy response in subtypes of hepatocellular carcinoma based on mitophagy
Source: Front Immunol. 2022 Oct 11;13:966167. doi: 10.3389/fimmu.2022.966167 (PMC9592915; doi:10.3389/fimmu.2022.966167)
Supplement: Supplementary file 1 [file Table_1.docx]

**Supplementary Table 1**. Information of online depository where our original data can be found.

| Depository | Github |
| --- | --- |
| URL | <https://github.com/pengdo9997/Supplementary.git> |
| Data content | 1. The expression matrix of peking union medical college hospital cohort 2. The expression matrix of HCC cell lines treated with and without cccp 3. The expression matrix of 8 pairs of HCC samples and peritumoral tissues |
