# Supplementary material for: Characterization of immune features and immunotherapy response in subtypes of hepatocellular carcinoma based on mitophagy
Source: Front Immunol. 2022 Oct 11;13:966167. doi: 10.3389/fimmu.2022.966167 (PMC9592915; doi:10.3389/fimmu.2022.966167)
Supplement: Supplementary file 2 [file Table_2.docx]

**Supplementary Table 2**. Primer sequences used for real-time polymerase chain reaction

| *ATG9A*-F | CTGCCCTTCCGTATTGCAC |
| --- | --- |
| *ATG9A*-R | CTCACGTTTGTGGATGCAGAT |
| *ATG12*-F | TTC CCC AGA CCA AGA AGT TG |
| *ATG12*-R | CAC GCC TGA GAC TTG CAG TA |
| *HRAS*-F | ATGACGGAATATAAGCTGGTGGT |
| *HRAS*-R | GGCACGTCTCCCCATCAATG |
| *MFN1*-F | TGGCTAAGAAGGCGATTACTGC |
| *MFN1*-R | TCTCCGAGATAGCACCTCACC |
| *NRAS*-F | TGAGAGACCAATACATGAGGACA |
| *NRAS*-R | CCCTGTAGAGGTTAATATCCGCA |
| *PGAM5*-F | TCGTCCATTCGTCTATGACGC |
| *PGAM5*-R | GGCTTCCAATGAGACACGG |
| *SQSTM1*-F | ATCGGAGGATCCGAGTGT |
| *SQSTM1*-R | TGGCTGTGAGCTGCTCTT |
| *TOMM5*-F | CGGAACTTTCTCATCTACGTGGC |
| *TOMM5*-R | ACCGTTCAGCTCAGTTCGAAGG |
| *TOMM22*-F | CAGTCCCCGGACGAATTGC |
| *TOMM22*-R | CGACAGGGTCTCATCTAGCTC |
| *GAPDH*-F | TGGTATGACAACGAATTTGG |
| *GAPDH*-R | TCTACATGGCAACTGTGAGG |
