# Supplementary material for: Characterization of immune features and immunotherapy response in subtypes of hepatocellular carcinoma based on mitophagy
Source: Front Immunol. 2022 Oct 11;13:966167. doi: 10.3389/fimmu.2022.966167 (PMC9592915; doi:10.3389/fimmu.2022.966167)
Supplement: Supplementary file 3 [file Table_3.docx]

Supplementary Table 3. Links to large files of our working sheets

| Data sheets | Links |
| --- | --- |
| Working sheet of TCGA | https://www.jianguoyun.com/p/DaeGyw8QoNnWChiXlMUEIAA |
| Working sheet of ICGC | https://www.jianguoyun.com/p/DXpWalsQoNnWChj4k8UEIAA |
| Source data of Figure4C | https://www.jianguoyun.com/p/DUPh9R4QoNnWChialMUEIAA |
